# Significance of Histopathology of Appendectomy Specimens: Analysis From a Teaching Hospital of Pakistan

**DOI:** 10.7759/cureus.50270

**Published:** 2023-12-10

**Authors:** Ammara Saif Ullah, Reemal Mushtaq, Haseeb Mehmood Qadri, Hasan Saeed, Muhammad Sheraz, Muhammad Faraz K Nizami, Saba Waheed, Momin Ijaz, Warda Fatima, Maha Saeed

**Affiliations:** 1 Surgery, Lahore General Hospital, Lahore, PAK; 2 Surgery, Jinnah Hospital, Lahore, PAK; 3 Surgery, Allama Iqbal Medical College, Lahore, PAK; 4 Surgery, Akhtar Saeed Trust Hospital, Lahore, PAK

**Keywords:** audit, alvarado, negative appendectomy, appendicectomy, appendectomy, appendix, biospy, histopathology

## Abstract

Background

Histopathology of a tissue specimen plays a crucial role in formulating the final diagnosis of any disease. It confirms whether the histopathological findings are in correspondence with the clinical diagnosis and thus suggests an optimal management plan. Standard surgical practices guide that every human tissue specimen must undergo postoperative tissue analysis unless indicated otherwise.

Objective

To determine the significance of histopathology in determining the final diagnosis of appendectomy specimens.

Materials and methods

This retrospective clinical study conducted in May 2022 included 100 patients operated for appendectomy from January 1, 2021, to December 31, 2021, in the emergency room of the Department of General Surgery, Unit-III, Lahore General Hospital, Lahore. Data were retrieved from patients' records and the picture archiving and communication system (PACS). A Google Forms-based *pro forma* (Google, Mountain View, CA) was generated to include the demographic details, clinical manifestations, and histopathology reports of the patients. Descriptive analysis was completed using a Microsoft Excel spreadsheet (Microsoft Corporation, Redmond, WA).

Results

Fifty-two patients were females out of the total 100. The mean age at presentation was 23.02 ± 12.02 years. Of the samples, 54% were not sent for histopathology. Among the remaining ones, 27% of cases were proven to be acute appendicitis. Alvarado score was 7-10 in 50% of patients. Other lesions proven by histopathology were appendiceal phlegmon (4%), perforated appendix (4%), mucocele (1%), carcinoid tumor (1%), tuberculosis (1%), and adenocarcinoma (1%).

Conclusions

Histopathological analysis is the gold standard for the tissue diagnosis of a disease. The high percentage of the samples not sent for histopathology is alarming since the appendix is not only a site for inflammatory pathologies but for neoplastic lesions as well. This practice depicts that the incidence of non-inflammatory pathologies is being ignored by healthcare professionals and there is a dire need to emphasize the significance of acquiring histopathology reports for the specimens of appendectomy in all circumstances.

## Introduction

Appendicitis is a frequently diagnosed pathology and an indication of appendectomy. It is defined as the inflammation of the inner lining of the vermiform appendix [1,2]. It is one of the most common surgical emergencies presenting with acute abdominal pain. Acute appendicitis is usually diagnosed clinically [3]. Many surgeons do not want the appendectomy specimens to undergo histopathological assessment, which can result in missed diagnoses and incorrect management.

Postoperative histopathology, conducted on specimens retrieved during surgery, is pivotal for the final diagnosis. It helps to confirm whether or not the clinical diagnosis, made on the basis of signs and symptoms, was correct and if the suspected lesion has been successfully resected [3,4]. This aids the clinician in deciding what further management the patient needs. It is fundamental surgical practice to always send the specimen for histopathology (unless clinically indicated otherwise) and not to rely on clinical assessment alone [4].

The histopathology findings directly influence the patient’s postoperative management and determine the need for any further treatment. Different findings upon histopathology include acute gangrenous appendicitis, neoplasia, diverticulitis, parasites, endometriosis, and various granulomatous diseases [4]. One can imagine the sinister impact of missing such diagnoses on the life of the patient. On the other hand, histopathology may reveal a normal appendix vermiformis, which necessitates further investigations to look for other pathologies. These diverse findings emphasize the importance of histopathology in the assessment of appendectomy specimens [1]. The Nepalese literature supports the notion of ordering histopathology of appendectomy specimens that are considered suspicious of malignancy intraoperatively [5]. Unfortunately, in Pakistan, histopathology reports are not taken in notice postoperatively, which poses a serious threat to the patient’s health and treatment. In our day-to-day practice in Lahore, Pakistan, it has been observed that healthcare professionals do not frequently send and follow the specimens for histopathological analysis once they retrieve them during a surgical procedure, and that specimen gets wasted in human disposal. This practice is certainly against surgical norms. Dafle et al. in 2020 suggested that systematic examination of all the surgically removed appendectomy specimens is crucial to confirm the pathology [6].

With these considerations in mind, the authors conducted a comprehensive study aimed at evaluating the practices of healthcare professionals at a tertiary care hospital in Lahore, a city considered as one of the healthcare hubs in Pakistan.

## Materials and methods

This retrospective cohort study was conducted from May 2, 2022, to May 31, 2022, at the Department of General Surgery, Unit-III, Lahore General Hospital, Lahore, Pakistan. Data of patients who presented to the emergency department from January 1, 2021, to December 31, 2021, were retrieved. Department of General Surgery, Unit-III, Lahore General Hospital issued approval SU-III/74/LGH, dated April 1, 2022. Departmental Ethical Approval was taken for this clinical audit from the Departmental Ethical Committee, after explaining the study design and its objectives. The study is consistent with the Declaration of Helsinki.

A Google Forms-based questionnaire pro forma (Google, Mountain View, CA) was generated, including the demographic details, clinical presentation, management, and histopathology reports. Histopathology reports were followed from patients' manual records and the picture archiving and communication system (PACS).

Inclusion criteria

Patients, irrespective of age or gender who underwent an appendectomy in the emergency, with complete file records available.

Exclusion criteria

Patients who underwent an appendectomy on an elective basis or with incomplete file records or missing information in PACS.

As it is an audit-based study, consecutive sampling was used to include all records of one year meeting the above-stated criteria, without calculating the sample size. Descriptive analysis was done using a Microsoft Excel spreadsheet (Microsoft Corporation, Redmond, WA).

Operational definitions

Patients were stratified into three groups having a likelihood of acute appendicitis, as shown in Table 1 [7].

**Table 1:** Groups of the likelihood of acute appendicitis as per the Alvarado scoring system.

Score range	Chances of appendicitis
1-4	Possible
5-6	Probable/equivocal
7-10	Predictable

## Results

**Figure 1:**
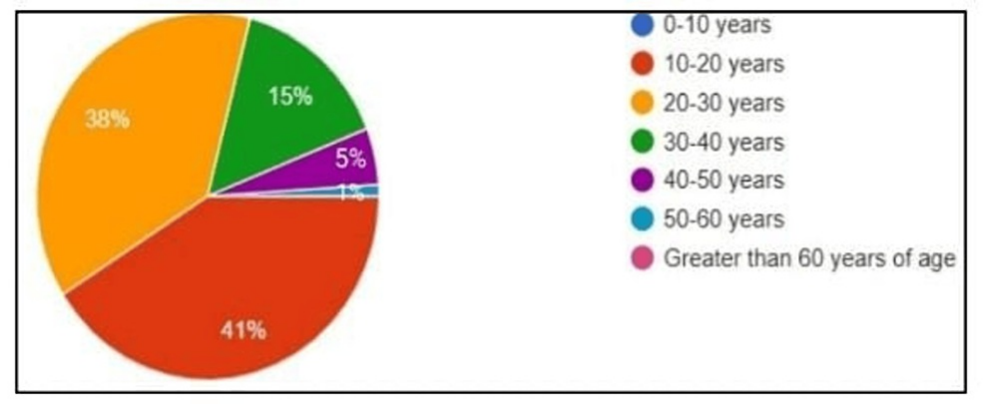
Stratification of included cases of acute appendicitis by age.

Data of 100 patients were collected, including 48% males and 52% females. The average hospital stay was 3.2 days, with a mean age of 23.02 ± 12.02 years. A majority of patients were from the age group of 10 to 20 years (Figure 1).

Of the patients, 95% were managed by open appendectomy using Gridiron incision and the other 5% underwent exploratory laparotomy with mid-line incision. Patients were majorly investigated by using ultrasound in 99% and computed tomography (CT) in 1% of cases only. The Alvarado scoring system was predictable of appendicitis in 50% of patients (Table 2).

**Table 2:** The Alvarado score range of included patients in terms of frequency.

Score range	Chances of appendicitis	Number of patients	Percentage of patients
1-4	Possible	14	14%
5-6	Probable/equivocal	36	36%
7-10	Predictable	50	50%

In observed cases, 54% of the samples were not sent for histopathology and 27% of cases were proven to be of acute appendicitis. However, other significant surgical pathologies found are stated in Table 3.

**Table 3:** Histopathological details of included cases in terms of percentages.

Histopathological details (N = 100)	
Types of cases	Percentage cases
Biopsy not sent	54%
Negative appendectomy	6%
Types of lesion	
Acute appendicitis	27%
Appendiceal phlegmon	4%
Perforated acute appendicitis	4%
Gangrenous appendicitis	1%
Mucocele	1%
Carcinoid tumor	1%
Tuberculosis	1%
Adenocarcinoma	1%

## Discussion

Acute appendicitis is a common surgical emergency that requires prompt diagnosis and treatment. Although clinical presentation and imaging play crucial roles in its evaluation; however, histopathology remains a cornerstone in confirming the diagnosis and guiding appropriate management. In this discussion, we will delve into the significance of histopathological examination of the appendectomy specimens and its impact on patient care.

The diagnosis of acute appendicitis is made mostly on the basis of clinical manifestations, with the accuracy of clinical diagnosis estimated between 76% and 92%, highlighting the importance of histopathology, as it is the gold standard for diagnosis [8-10]. Histopathological examination may disclose additional pathologies that may not be evident intraoperatively [10].

We collected the data of 100 patients whose provisional diagnosis of acute appendicitis was made. It showed slight preponderance to female gender as it affected 52% of females and 48% of males. This finding does not exactly coincide with the findings of Sharma et al., who found that males are affected more than females with a percentage of 68% and 32%, respectively [10]. The reason behind slight female predominance is yet to be unveiled but one possible explanation is we only considered the Alvarado scoring system to diagnose acute appendicitis, which does not take into account gender just like the RIPASA (Raja Isteri Pengiran Anak Saleha Appendicitis) scoring system in which the male gender is given a score of 1 compared to females given a score of 0.5. Those studies that concluded male dominance, as discussed above, considered the RIPASA scoring system as a screening tool for acute appendicitis [9]. Thus more males were diagnosed with acute appendicitis compared to females. Our setup used the Alvarado scoring system that does not consider gender during screening [9].

The average hospital stay was 3.2 days, which is more than 2.57 days, as described by Chan et al. [3]. However, this stay is less than that described by Nazir et al. in 2019, which concluded the average stay in open appendectomy to be 4.18 ± 0.77 days [11].

The mean age was 23.02 ± 12.20 years in our study, which is in accordance with the study conducted by Muhammad et al. and Sharma et al. where the mean age in the appendectomy group was 32 ± 14 years and 20-30 years, respectively [10,11].

The Alvarado scoring system was used for this study, which showed a predictability of 50%. A local study showed that the Alvarado score of 6 or more has a diagnostic accuracy of 82.9% in predicting acute appendicitis in children [2]. In a study by Sana et al., the Alvarado score, modified Alvarado score, RIPASA, and Lintula scales were also used and the RIPASA score was found to be more sensitive and specific compared to the Alvarado score because it takes into account more information than the modified Alvarado score, i.e., gender and age, and it also rules out urinary causes of right iliac fossa pain. Hence, it proves to be more beneficial [9].

Ultrasound of the abdomen was implied as the primary mode of investigation (99%), while a CT scan of the abdomen was used in only one patient. Ultrasonography scan (USS) is the first-line diagnostic modality as stated by Chan and colleagues [3]. USS has a specificity of 87% and a sensitivity of 90%. The reason behind being the first choice is its easy accessibility and being favorable to rule out gynecological causes. However, the only drawback of USS is that it is user-dependent. CT scan is also a useful and helpful modality to diagnose those cases that remained undiagnosed after USS, but interestingly, Chan et al. described that those patients who underwent CT scan had increased incidence of perforation. This is because of the temporal delay related to arranging a CT scan [3].

In our study, all patients were managed surgically. Of the patients, 99% were managed by an open appendectomy using a Gridiron incision and the other 5% underwent exploratory laparotomy using a midline incision. In observed cases, either 54% of samples were not sent for histopathology or were not followed. Ignorance of tracking patient reports or under-emphasis on the significance of histopathology could be the reasons.

We concluded that 27% of cases were proven to be acute appendicitis on histopathology, which is in contrast to other studies that showed the rates of acute appendicitis to be 62.64%, 64%, and 56% [8,10,12]. This low incidence can be attributed to the lack of histopathology assessment in most of cases. The suggested rate of negative appendectomies is 20% in the existing English scientific literature to avoid the neglect of appendicitis and the development of possible occurrence of complications [13]. The occurrence of negative appendectomies was 6% at our center, in accordance with the study by Emre and colleagues (6%) and Sharma et al. where it was 5.7% [1,10]. This is in contrast to the study by Maryam et al. where the rate of negative appendectomies was 3.82% [12]. The histopathology report of our patients demonstrated that 4% of the lesions were appendiceal phlegmon and 4% were of perforated appendix. This is in contrast to a study by Kepil et al. where the rates were found to be 37-38% for appendiceal phlegmon and 2-6% for perforated appendix [14]. Again, the very low rate of appendiceal phlegmon in our study can be attributed to the lack of histopathology reports in most of the cases.

We found out that mucocele was present in 1% of the cases, which is exactly similar to the findings of a study by Emre et al., i.e., 1% [1]. However, in contrast to this, the analysis by Sharma et al. described a relatively low incidence of mucocele, i.e., about 0.4% [10]. Moreover, carcinoid tumor also constitutes 1% of the diagnosed cases, similar to the study by Maryam et al., which showed the occurrence rate of carcinoid tumor to be 1.76% [12]. Contrary to our results, the study published by Emre et al. in 2013 showed the percentage of carcinoid tumors about 11% [1]. There are some other conditions diagnosed on histopathological analysis of specimens, such as gangrenous appendix with an incidence of 1%. Although extremely uncommon of all the gastrointestinal neoplasms, adenocarcinoma appendix still accounts for 0.5% of all gastrointestinal tumors [15]. The most worrisome diagnosis of adenocarcinoma, made on microscopic examination of specimens, was also present in 1% of cases, which is almost similar to the results of Sharma et al. where 1.4% of cases involved neoplastic lesions [10].

Limitations

Due to a limited sample size, unicentric and retrospective nature, the results cannot be generalized to a larger group of population.

Clinical recommendations

Appendectomy specimens should be sent for histopathological examination to find out the exact cause of appendicitis, which, in some cases, could be carcinoid tumors, adenocarcinomas, or parasitic infections, which may require further treatment. This situation is alarming because it may endanger the lives of the patients as the undiagnosed conditions can progress unchecked, potentially leading to delayed treatment and a poor prognosis.

Nonoperative treatment should be opted for if the Pediatric Appendicitis Score (PAS) is 7, the duration of symptoms is shorter, and there is no appendicolith or complex peri-appendiceal fluid on ultrasound. RIPASA score is the most sensitive and specific scoring system to diagnose acute appendicitis.

## Conclusions

The existing literature underscores the role of postoperative histopathological examination of the specimen in the management of acute appendicitis. It does not only confirm the diagnosis of acute appendicitis but also helps to rule out other differential diagnoses, including potentially fatal conditions, such as malignancy and tuberculosis. Moreover, histopathological examination also plays a crucial role in identifying negative appendectomies, which prompts surgeons to explore alternative causes of acute abdominal conditions and adapt their clinical practices accordingly. The data highlight the importance of routinely incorporating histopathological assessment into appendectomy protocols so that we do not miss serious pathologies of the appendix. In addition to that, histologically negative appendectomies indicate the need to implement new scoring systems and additional diagnostic modalities before proceeding with an appendectomy.
